# *Fraxinus excelsior* updated long-read genome reveals the importance of MADS-box genes in tolerance mechanisms against ash dieback

**DOI:** 10.1093/g3journal/jkaf053

**Published:** 2025-03-20

**Authors:** Sara Franco Ortega, James A Bedford, Sally R James, Katherine Newling, Peter D Ashton, David H Boshier, Jo Clark, Susan E Hartley, Andrea L Harper

**Affiliations:** Centre for Novel Agricultural Products, Department of Biology, University of York, York YO10 5DD, UK; Centre for Novel Agricultural Products, Department of Biology, University of York, York YO10 5DD, UK; Biology Technological Facility, Department of Biology, University of York, York YO10 5DD, UK; Biology Technological Facility, Department of Biology, University of York, York YO10 5DD, UK; Biology Technological Facility, Department of Biology, University of York, York YO10 5DD, UK; Department of Plant Sciences, University of Oxford, Oxford OX1 3RB, UK; Future Trees Trust, Coleshill SN6 7PT, UK; Centre for Novel Agricultural Products, Department of Biology, University of York, York YO10 5DD, UK; School of Biosciences, University of Sheffield, Sheffield S10 2TN, UK; Centre for Novel Agricultural Products, Department of Biology, University of York, York YO10 5DD, UK

**Keywords:** ash, *Fraxinus excelsior*, genome, *Hymenoscyphus fraxineus*, tolerance, diversity panel, dormancy, associative transcriptomics, Oxford Nanopore Technologies

## Abstract

Ash dieback caused by the fungus *Hymenoscyphus fraxineus* has devastated the European ash tree population since it arrived in Europe in 1992. Great effort has been put into breeding programs to increase the genetic diversity of ash trees and find heritable genetic markers associated with resistance, or tolerance mechanisms, to ash dieback. To facilitate identification of molecular markers, we used Oxford Nanopore Technologies combined with Illumina sequencing to obtain an accurate and contiguous ash genome. We used this genome to reanalyze transcriptome data from a Danish ash panel of 182 tree accessions. Using associative transcriptomics, we identified 175 gene expression markers, including 11 genes annotated as dormancy MADS-box transcription factors which are associated with ash bud dormancy, flowering, and senescence. We hypothesize that tolerant trees both break dormancy earlier in the year by increasing the expression of flowering-related SOC1 MADS-box and reducing the expression of SVP-like MADS-box, whilst also accelerating senescence by increasing the expression of JOINTLESS MADS-box genes. DNA methylation differences in the promoters of MADS-box genes between 1 tolerant and 1 susceptible tree indicate potential epigenetic regulation of these traits.

## Introduction

Common ash (*Fraxinus excelsior*) is a medium-sized deciduous tree with a wide distribution throughout all temperate zones of Europe. Ash is vulnerable to attack by many pests, for example, the emerald ash borer (*Agrilus planipennis*), a beetle native to Asia, whose larvae feed in the phloem, threatening the survival of the tree. However, since the first observation in 1992 in Poland, ash dieback caused by the ascomycete fungus *Hymenoscyphus fraxineus* (syn. *Chalara fraxinea*) has devastated European ash trees, killing over 90% of trees, reaching the UK in 2012 (Freer-Smith and Webber 2017). Ash dieback causes wilting and necrotic lesions on leaves, and later dot diamond-shaped lesions on the stems. Eventually, the disease kills the majority of trees, with only 5% of trees exhibiting low susceptibility to ash dieback. The teleomorph form of the fungi develops on the fallen rachises from leaves infected during the previous year and produces airborne ascospores reaching inocula of more than 100 spores m^3^, allowing transmission to other trees (Chandelier *et al*. 2014).

Due to both long generation and production cycles, forest tree breeding programs emphasize the maintenance of genetic variability whilst selecting to improve particular traits (Neale and Kremer 2011). Genomics-assisted breeding offers new opportunities to exploit wild tree populations whilst maintaining their genetic diversity (Migicovsky and Myles 2017). Genome-wide association studies can be used to determine loci responsible for a trait (resistance, tolerance, or susceptibility) and their size effect on the phenotype. Previously, more than 3,000 single-nucleotide polymorphisms (SNPs) were associated with low vs high ash dieback disease scores, and 61 were associated with the biotic stress response in other plant species. Prediction models using these data were able to estimate the health score of the tree with 90% accuracy (Stocks *et al*. 2019). However, this analysis was performed on a relatively low-contiguity ash genome reference (89,487 scaffolds with N50 of 104 kbp) (Sollars *et al*. 2016), which could cause an overestimation of marker-trait associations due to inaccuracies across regions with high numbers of undetermined nucleotides (∼17% of this genome reference).

Obtaining an accurate, contiguous genome is a key step for downstream analysis. Here, we report the assembly of a new high-contiguous genome for European ash, and an assessment of the genetic diversity in a Danish ash population with a high incidence of ash dieback (for more information about the panel, please refer to Harper *et al*. 2016). We used Oxford Nanopore Technologies (ONT) to generate long sequence reads combined with Illumina data, to obtain a more contiguous and complete ash genome. (Fleck *et al*. 2024) have already reported that ONT data is sufficient for assembling *Fraxinus* spp. genomes as they obtained contiguous genomes of *Fraxinus americana*, *Fraxinus nigra*, and *Fraxinus pennsylvanica*. However, in this work, we benefit from previous Illumina data that we used to polish the genome and for de-novo annotation. To assess the utility of this assembly, we analyzed mRNA-Seq data via associative transcriptomics (AT) analysis, which identifies molecular markers associated with a trait measured across a diversity panel, and has been used previously to identify gene sequence and expression variants associated with ash dieback damage using a Danish population (*n* = 182) (Harper *et al*. 2016). Previously, Harper *et al*. (2016) identified 1 SNP and 13 gene expression markers (GEMs) associated with low and high disease tolerance to ash dieback. Both the SNP and the top 2 GEMs were members of the MADS-box transcription factor family and could predict 21 and 28% of the phenotype of other accessions. In this manuscript, we performed an AT analysis using the updated *F. excelsior* gene annotations and the same Danish population, confirming the importance of some MADS-box transcription factors such as *SUPPRESSOR OF OVEREXPRESSION OF CONSTANS1* (SOC1) or *SHORT VEGETATIVE PHASE* (SVP)-like genes, identified by subsequent phylogenetic analysis, in tolerance/susceptibility to ash dieback.

A great additional advantage of using ONT technologies is the possibility of calling methylated sites on the genome. In plants, cytosine DNA methylations (5mCs) occur in 3 sequence contexts CpG, CHG, and CHH (where H = A, C, or T). Methylation of cytosine plays an important role in biological processes, for example, in gene expression regulation, silencing of transposable elements, or stress response, as differential DNA methylation can be related to selective expression of defense-related genes (Arora *et al*. 2022). Understanding differences in methylation between tolerant and susceptible ash trees could help to understand the basis of these phenotypes. For the first time, we have linked the expression of genes associated with ash dieback tolerance to significant differences in the DNA methylation frequency of the promoters of GEMs identified by the AT analysis.

### Materials and methods

#### DNA extraction and sequencing

DNA was obtained from the leaves of a grafted *F. excelsior* tree which was used for an earlier ash genome assembly (isolate 2451S; Sollars *et al*. 2016), which was provided to us by Future Trees Trust. The parent tree located at Paradise Wood, Oxfordshire, UK, was produced by the self-pollination of a hermaphroditic *F. excelsior* tree growing in woodland in Gloucestershire, UK, (52.020592, −1.832804) in 2002 as part of the FRAXIGEN project (Fraxigen 2005).

Leaves were ground to a powder in liquid nitrogen, before DNA was extracted using a high molecular weight gDNA extraction protocol. Briefly, ground leaf material is incubated with Calson buffer (100 mM Tris–HCl pH 9.5, 2% CTAB, 1.4 M NaCl, 1% PEG 8000, 20 mM EDTA, and 0.25% β-mercaptoethanol) containing proteinase K at 65°C for 30 min with intermittent mixing, followed by an addition of RNaseA for a further 30 min incubation. An initial crude extraction was performed with 1 volume chloroform, and the DNA precipitated with 0.7 volumes isopropanol. The resultant DNA was then subject to a further purification using QIAGEN genomic-tips, according to the protocol developed by Vaillancourt and Buell (2019). Extracted DNA was quantified with Qubit and Nanodrop prior to sequencing. Libraries were prepared using the SQK-LSK109 ligation sequencing kit, using extended incubations for nick repair, end preparation and ligation steps, and each sample run on a single PromethION R9.4.1 (FLO-PRO002) flow cell. Sequencing was performed on a PromethION 24 running MinKNOW software version 3.6.1, and highest accuracy basecalling performed using Guppy basecaller version Guppy basecalling software version 3.2.8 (ONT).

#### Genome assembly

Initially, the chloroplast and mitochondrial genomes were assembled using CANU (version 2.1.1) (Koren *et al*. 2017) and polished using RACON version 1.4.20 and MEDAKA version 1.0.3. (Vaser *et al*. 2017) (Supplementary Text 1). CANU was then used to assemble the genome with the remaining unmapped reads.

To curate the heterozygous genome, PURGE HAPLOTIGS (Roach *et al*. 2018) was used to reassign allelic contigs and obtain a haploid assembly. The curated assembly was plotted to calculate the coverage and find 0.5× and 1× coverage contigs using the histogram cutoffs: a low point of 3, a midpoint of 45, and a high cutoff of 390. All the contigs with abnormally high or low coverage according to the histogram were removed and those with an alignment score greater than 80% were also marked for reassignment as haplotigs (option -a 80 in the purge function). Previously published Illumina data (ERR1399574; Sollars *et al*. 2016) was used to polish the curated assembly using PILON version 1.24 (Walker *et al*. 2014).

Benchmarking Universal Single-Copy Orthologs (BUSCO version 4.0; Manni *et al*. 2021) was used to assess the completeness of the genome against the embryophyte and eudicot datasets. Statistical analyses were also performed with QUAST v 5.0.2 (Gurevich *et al*. 2013).

#### Genome annotation

To annotate the genome, repetitive elements were identified using REPEATMODELER (version 2.1) and REPEATMASKER (version 2.1; Flynn *et al*. 2020). BRAKER (version 1.9; Brůna *et al*. 2021) was used to predict the gene models in the masked genome. Protein data from the Viridiplantae (downloaded November 2021; https://v100.orthodb.org/download/odb10_plants_fasta.tar.gz; Zdobnov *et al*. 2021) was used in combination with the previously published mRNA-Seq data from roots (ERR1399494), cambium (ERR1399492), leaves (mother tree and 2461s, ERR1399495, ERR1399573), and flowers (ERR1399493). TSEBRA (Gabriel *et al*. 2021) with default configuration was used to combine the results of both proteins and mRNA-Seq predictions. Annotations were filtered by structure and function using GFACS (Version 1.1.2; Caballero and Wegrzyn 2019) and ENTAP (Hart *et al*. 2020). EGGNOG (Huerta-Cepas *et al*. 2019; Cantalapiedra *et al*. 2021) was used for the functional characterization of the gene models. Transposable elements (TE) and tandem repeats were identified using the Extensive de-novo TE Annotator (EDTA; Ou *et al*. 2019). We compared the gene models predicted in this new assembly against the cds models reported by Sollars *et al*. (2016). We identified the genes with the highest homology between both genomes by first creating a database using the old genome followed by BLASTn with the new cds models as a query and filtering for a single match (-max_target_seqs 1). Gene ontology enrichment was performed using the package topGO in R assessing the molecular function, cellular components and biological processes of the new cds models that did not have high similarity with the predicted genes in the genome reported by Sollars *et al*. (2016).

#### DNA methylation

NANOPOLISH (v 0.13.2; Simpson *et al*. 2017) was used to identify methylated CpG sites. DNA regions with a cytosine followed by guanine from 5′ to 3′ direction were identified as well as the frequency of methylation in the whole genome. The frequency was only calculated when the log_lik_ratio (log like methylated–log like unmethylated) had a positive value, supporting methylation.

#### Pseudochromosome construction

A closely related species with a chromosome-level genome assembly can be used as a reference to construct pseudochromosomes from the contigs. We used the chromosome-level *F. pennsylvanica* genome (Huff *et al*. 2022) in NTJOIN (version 4.2.1; Coombe *et al*. 2020) with k = 32 and w = 32, *n* = 2, mkt = true and no_cut = true options. NUCMER 3.1 (Kurtz *et al*. 2004) was then used for mapping (options -c 500 -b 500 -l 100 -maxmatch) between the *F. pennsylvanica* chromosomes and the *F. excelsior* pseudochromosomes. Files were later filtered (delta-filter function) to only maintain the synteny regions with identity ≥ 90% and with a minimum length of 100 bp.

Circlize package in R was used to create a chord graph of syntenic regions with identity > 90% and using a minimum length of 100 bp. A circular plot of the genome was then performed using the Circlize package in R, showing the position on the pseudochromosomes of the contig and genes, GC content, methylated frequency, coverage and long terminal repeat (LTR) transposable elements.

Mapping between genes (using the coding sequence) of both species was carried out using the Synteny Imaging tool (SYNIMA; Farrer 2017) using OrthoFinder.

#### Population structure analysis of Danish trees

To investigate the genetic diversity of *F. excelsior* in Denmark, we used previously published data (*n* = 182, Harper *et al*. 2016). The mRNA-Seq data was mapped using STAR 2.7.10 (Dobin *et al*. 2013) against the new genome assembly, and variant calling was performed using GATK version 4.2.4.0 using HaplotypeCaller. VCFTOOLS version 0.1.16 (Danecek *et al*. 2011) was used to merge variants for the individuals of each panel, and to filter out genotypes called below a minimum allele count = 5, minimum number of alleles = 2, maximum missing data 25% (across all individuals), SNPs with >3 alleles, and quality < 30. After filtering, the samples were merged using VCFTOOLS. Annotation of the variants was done with SnpEff version 5.1 (Cingolani *et al*. 2012b) and filtered with SnpSift (Cingolani *et al*. 2012a) to only keep SNPs located on exons. The vcf file was converted to geno format using VCFTOOLS and the ped2geno function (LEA package) in R. PSIKO v2 (Popescu and Huber 2015) was used to infer population stratification.

#### AT

To assess the validity of the new genome, we repeated the AT analysis of ash dieback disease damage traits in the Danish panel (Harper *et al*. 2016). To identify any differences in the gene expression, TPMs (transcripts per million) of the mRNA-Seq data from the Danish panel were calculated using salmon (version 0.8; Patro *et al*. 2017). Salmon counts were input into R 4.3.3 with tximport (Soneson *et al*. 2016) to obtain TPMs. Regression analysis was then conducted to compare the expression (TPMs) of the 182 trees of the Danish panel against the ash dieback disease damage scores (for more information about how damage score was measured, refer to Harper *et al*. 2016). The analysis included multiple test corrections using false discovery rate (FDR) with Benjamini–Hochberg adjustment and Bonferroni threshold to reduce the likelihood of identifying false positives. We identified GEMs by setting FDR ≤ 0.05.

#### Phylogeny of MADS-box genes

To identify the role of MADS-box genes, classified as GEMs, we retrieved the amino acid sequence of the MADS-box genes with FDR < 0.05 in the AT analysis, and the sequence of another 63 MADS-box sequences from *Arabidopsis thaliana*, *Pyrus pyrifolia*, and *Prunus mumus* downloaded from GenBank (NCBI, National Center for Biotechnology Information). Sequences were then aligned using MAFFT (version v7.490) (Katoh and Standley 2013), trimmed with TRIMAl (version 1.4 rev15; Capella-Gutiérrez *et al*. 2009) and the maximum likelihood phylogenetic tree was created using IQ-TREE (version 2.2.0-beta; Nguyen *et al*. 2015). Maximum likelihood was performed on 118 amino acids with the Q.insect + G4 chosen automatically according to the Bayesian information criterion. The Interactive Tree of Life online tool (I-TOL version 6.5.8) was used to visualize the tree.

#### Methylation profile of tolerant and susceptible trees

With the aim of identifying potential epigenetic regulations that might control the expression of GEMs, we extracted DNA was using the method of Workman *et al*. (2018) from a pair of Danish trees previously described in Harper *et al*. (2016); association panel tree Ash66, and prediction panel tree DNASH35, which had ash dieback disease damage scores of 0 and 75%, respectively. ONT library preparation, sequencing, basecalling, and methylation calling were performed as described above. NANOPOLISH was used to call methylation as described above. Methylation was assessed across different genomic locales (gene bodies, 1 kbp upstream and 1 kbp downstream regions) of all the genes in the genome. Plots were generated using ggplot2 in R representing the mean methylation frequency (20 intervals each representing 5% of the length of the gene body or the mean in each of the 20 intervals representing 50 bp of the upstream and downstream regions). To identify potential epigenetic regulation of GEMs, we assessed the methylation frequency in the upstream region (promoters) of 3 GEMs with lower FDR (Fe_g19663, Fe_g22999, and Fe_g40353). We first removed outliers with identify_outliers function in R and then compared methylation frequency between the susceptible and tolerant trees to ash dieback using the function t_test in R. To associate DNA methylation changes with gene expression, we also assessed and plotted using ggscatter and reg.line in R, the expression of these 3 GEMS across the Danish panel.

### Results and discussion

#### A high-quality *F. excelsior* genome assembly

We generated >49 billion bp in 16,511,805 reads (41.1% Q20, 10.5% Q30) with an average read length of 2,990.7, equating to >47× coverage of the previously reported 877 Mbp ash genome (Sollars *et al*. 2016). After chloroplast and mitochondrial assembly (Supplementary Text 1), the remaining reads (14,785,500; 40 billion bases; 45.53× coverage) were assembled into 4,353 contigs and, after polishing with Illumina reads, a genome with a total length of 866,153,998 bp, N50 = 336,336 (minimum length 1,121 bp and maximum length 2,511,904) and a 34.22% average GC content was obtained. Although this assembly is 11 Mbp smaller than the previous assembly (Sollars *et al*. 2016), we hypothesize that it is due to far fewer undetermined “N” bases in the new assembly (0% compared to 17%), which can lead to overestimation of genome size. The BUSCO completeness score against the eudicots database was 81.7%, consisting of 68.5% single-copy and 13.2% duplicated BUSCOs. Against the embryophyte database, the completeness score was 85.1% consisting of 71.6% single-copy and 13.5% duplicated (fragmented: 6.4%, missing: 8.5%, *n*: 1,614).

The genome was annotated using a combination of mRNA-Seq data and protein gene model predictions. In total 43,392 gene models were obtained. Out of these, 15,245 were mono-exonic and 28,147 multi-exonic with a median of 4 introns each. The median gene size, coding sequence size and exon size was 1,181, 549, and 114 bp, respectively. Functional annotations were obtained for 39,864 of those gene models. The number of gene models increased by 12% compared with the previous assembly (38,852 protein-coding genes and 50,743 transcripts (Sollars *et al*. 2016)). We found that 38,588 new gene models had high homology to 26,078 old gene models (with multiple correspondence between 1 new cds model and several old cds models). The new gene models with no similarity (4,804) were assessed for their function by performing a gene ontology enrichment (Supplementary Table 1). This analysis showed that they matched with multiple functions and processes including retrotransposition, defense response, signal transduction, or RNA-templated DNA biosynthetic process. These differences could be explained by the advancement in predicting new gene models by programs like BRAKER.

TEs accounted for 50.15% of the genome (previously reported as 35.9% in Sollars *et al*. (2016)), with class I TEs having LTRs in almost 30% of the genome and class II divided into terminal inverted repeats (TIR) and non-TIRs (lacking the TIR structure) in 13.8 and 6.5% of the genome, respectively (Table 1). The level of cytosine C5 methylation was 71% (median), with a similar level of methylation in all pseudochromosomes and a median GC content of 43.4% (Supplementary Fig. 1b).

**Table 1.** Transposable elements (long terminal repeat, considering the 4,353 contigs and genome size of 866,153,998 bp.

Type	Class	Count	Number of bases masked	% masked
**LTR**	Copia	139,021	1.14E^+08^	13.18
	Gypsy	59,818	5.66E^+07^	6.54
	Unknown	130,001	8.78E^+07^	10.14
**TIR**	CACTA	73,048	2.44E^+07^	2.82
	Mutator	158,792	4.92E^+07^	5.68
	PIF_Harbinger	72,787	1.38E^+07^	1.59
	Tc1_Mariner	14,873	4.39E^+06^	0.51
	hAT	66,539	2.75E^+07^	3.18
**Non**-**TIR**	Helitron	157,970	5.65E^+07^	6.52
**Total**	Interspersed	872,849	4.34E^+08^	50.15

Using the *F. pennsylvanica* assembly (Huff *et al*. 2022), we assessed the synteny of the new *F. excelsior* genome and assigned the contigs into 23 pseudochromosomes covering 98.8% of the genome size (Supplementary Fig. 1a, Table 2), but adding undetermined nucleotides to the assembly (N's per 100 kbp = 33,878.9). Although some small contigs did not assemble within the pseudochromosomes, they only accounted for 1.2% of the total genome size and were likely discarded by NTJOIN, probably due to the small window used for minimizers (21 bp) and the different length of repetitive regions in the genomes or by local misassembles (Table 1). A total of 19,063 genes were identified as orthologues between *F. pennsylvanica* and *F. excelsior*, respectively (out of a total of 35,470 and 43,392 genes in the *F. pennsylvanica* and *F. excelsior* genomes, respectively) (Supplementary Table 2).

**Table 2.** Pseudochromosome sizes and number of contigs (4,353) were obtained in the new *F. excelsior* genome assembly.

Scaffold	Pseudochromosome	Number of contigs/scaffold	Size pseudochromosome considering only contigs (bp)	Size pseudochromosomes (bp)
**ntJoin11**	Fe_Chr01	278	57,049,569	86,871,061
**ntJoin3**	Fe_Chr02	424	49,314,643	74,385,309
**ntJoin7**	Fe_Chr03	173	37,946,013	61,580,219
**ntJoin17**	Fe_Chr04	166	41,423,499	63,362,762
**ntjoin15**	Fe_Chr05	215	46,945,204	70,449,272
**ntJoin20**	Fe_Chr06	166	38,821,743	57,332,361
**ntJoin19**	Fe_Chr07	138	30,142,704	47,976,435
**ntjoin1**	Fe_Chr08	153	35,917,803	55,280,346
**ntJoin4**	Fe_Chr09	165	40,278,964	60,937,593
**ntJoin2**	Fe_Chr10	175	36,769,960	56,950,872
**ntJoin5**	Fe_Chr11	135	30,206,494	48,899,062
**ntJoin12**	Fe_Chr12	18	43,071,336	64,260,172
**ntJoin6**	Fe_Chr13	176	39,691,603	58,994,051
**ntjoin8**	Fe_Chr14	161	36,996,715	52,400,938
**ntJoin16**	Fe_Chr15	186	35,541,244	51,705,577
**ntJoin18**	Fe_Chr16	126	27,257,267	42,313,996
**ntJoin0**	Fe_Chr17	205	46,292,443	65,972,964
**ntJoin9**	Fe_Chr18	133	30,323,337	45,688,329
**ntJoin22**	Fe_Chr19	140	30,209,446	48,559,296
**ntJoin21**	Fe_Chr20	95	26,387,313	39,753,493
**ntJoin13**	Fe_Chr21	149	33,344,042	47,622,639
**ntJoin10**	Fe_Chr22	121	26,241,128	41,519,084
**ntJoin14**	Fe_Chr23	153	34,940,498	50,364,800
**Not assigned**		507	11,041,030	

Overall, with the use of long-read sequencing technologies, we improved the contiguity and completeness of the previous ash genome based on a short-read assembly (Sollars *et al*. 2016) and reported a high level of TEs (>50%), methylation levels along the genome, and a 12% increase in the number of gene models. We also reported a chloroplast assembly with a larger size (192 kbp) than previous chloroplast genomes in other *Fraxinus* species such as *F. pennsylvanica*, which had a reported chloroplast genome of 155 Kb (Yi *et al*. 2019). Chloroplast inverted repeats are usually misassembled, or the boundaries of these regions are not always accurately reconstructed: this could be the case with the chloroplast of the ash genome. Many problems can be also faced in the complex mitochondrial genome due to the large sizes and multiple dynamic structures including linear, branched, or circular (Bendich 1996), which could explain the slight oversize of the mitochondrial genome (592 kbp) compared with the 581 kbp reported previously (Sollars *et al*. 2016).

#### Population genetic analysis to unravel ash dieback tolerance mechanisms

We first mapped Illumina reads from the Danish population to the new genome reference (the average mapping of the Danish was 84.5%; Supplementary Table 3). The Danish panel population structure was also assessed using PSIKO (Supplementary Table 4), based on the membership coefficient (>60%).

**Fig. 1.**
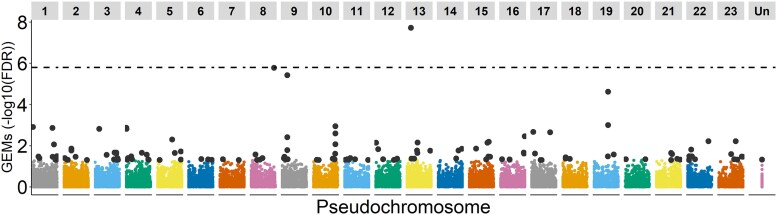
Gene expression markers (GEMs for ash dieback disease score) along the pseudochromosomes. The dotted line represents the Bonferroni cutoff, with only Fe_g19663 passing the threshold. Gray dots represent the GEMs with FDR < 0.05. “Un” represents the contigs that have not been assembled into pseudochromosomes.

A total of 175 GEMs with FDR ≤ 0.05 (Fig. 1, Supplementary Table 5) were identified using AT. Out of those, only Fe_g19663 passed the Bonferroni threshold, followed by Fe_g22999 and Fe_g40353 (Bonferroni > 0.05 and FDR < 0.05), all of which were identified as MADS-box genes. The gene previously reported as a cDNA-SNP marker for ash dieback susceptibility (Harper *et al*. 2016), had a high similarity (99%) with the CDS of the Fe_g18379 (FDR = 2.41E^−05^).

**Fig. 2.**
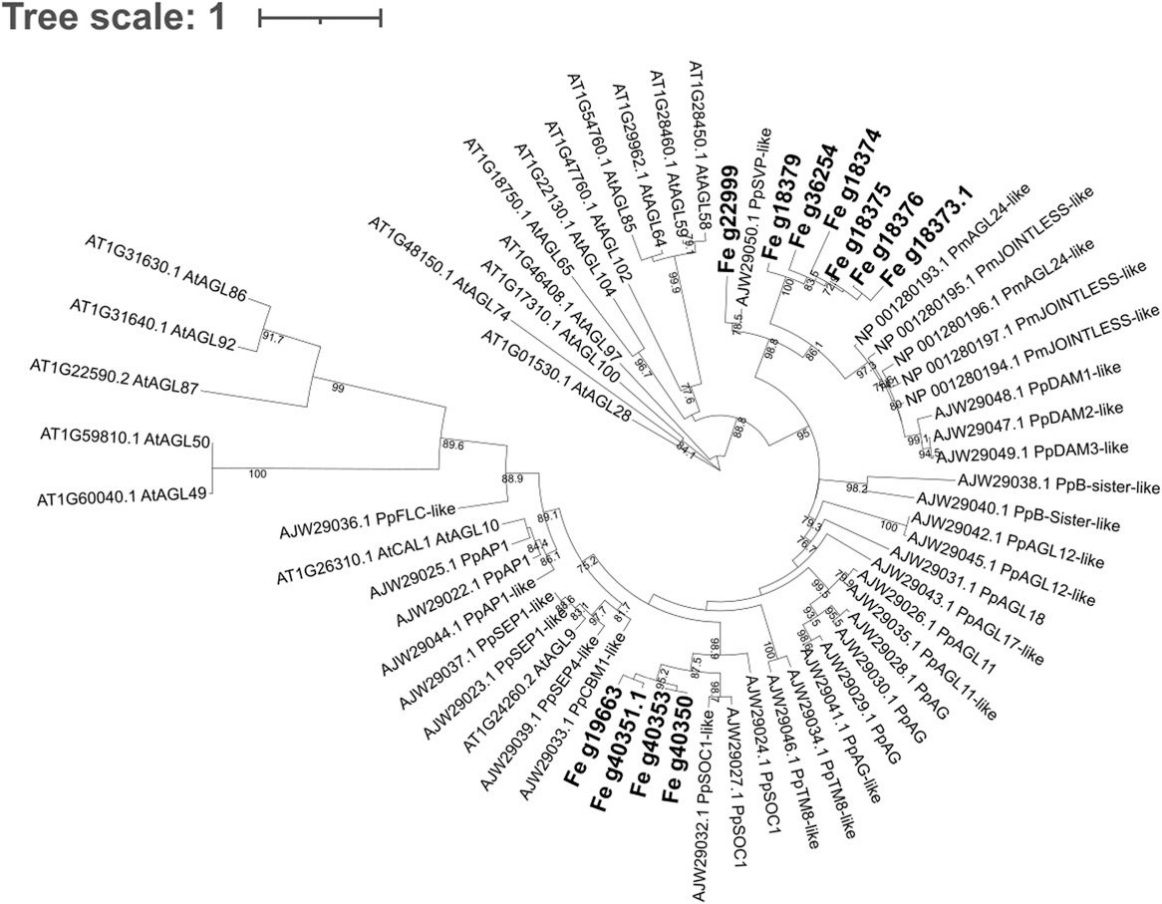
Maximum likelihood tree of the 11 MADS-box identified as GEMs. Bold genes are the MADS-box genes identified in the AT analysis and associated with susceptibility to ash dieback. The tree includes other MADS-box sequences from *Arabidopsis thaliana* (At), *Pyrus pyrifolia* (Pp), and *Prunus mumus* (Pm) downloaded from GenBank (accession number in the names). Bootstraps (from 70 to 100) are included in branches.

A total of 11 MADS-box genes were identified within the 175 significantly associated GEMs (FDR < 0.05). To better understand the role of these GEMs, we inferred a phylogenetic tree by creating a multi-locus sequence alignment with 63 sequences from *A. thaliana*, *P. pyrifolia*, and *P. mumus*, obtaining a total of 118 amino acids after trimming. After the phylogenetic analysis, the GEMs were identified as likely orthologues of the SOC1-like MADS-box (Fe_g19663, Fe_g40350, Fe_g40353, and Fe_g40351.1), and the dormancy MADS-box JOINTLESS (Fe_g18379, Fe_g36254, Fe_g18374, Fe_g18375, Fe_g18373.1, Fe_g18376) (Fig. 2). SOC1 has previously been identified as a promoter of flowering (Dorca-Fornell *et al*. 2011) whilst JOINTLESS has been associated with senescence (Nakano *et al*. 2015). Fe_g22999 was identified as SVP-like, a gene that in *Arabidopsis*, delays flowering by repressing floral regulators such as SOC1 (Li *et al*. 2008).

The high representation of MADS-box genes in the GEMs suggested a link between the phenology of the tree, such as dormancy, flowering and senescence, and susceptibility to ash dieback. In general, woody perennial plants modulate their seasonal phenology, growth, and dormancy cycles, according to environmental conditions. Bud flush starts with warmer temperatures and increasing daylight (in the UK from April to June), and later, the tree reaches the vegetative growth stage where it will reach its full canopy (June–August). With the reduction of daylight and colder nights in autumn (September–November), the trees stop growing and start forming buds, activating leaf senescence and abscission, followed by endodormancy and cold acclimation (November–March) (Ding and Nilsson 2016). After endodormancy, when the plant has accumulated enough chill, it gradually shifts to ecodormancy and if the conditions are favorable, the floral buds break (Alburquerque *et al*. 2008,Wang *et al*. 2020). On the other hand, *H. fraxineus* overwinters in the leaf litter on the ground. The wind-dispersed ascospores infect ash trees during the tree's vegetative growth stage (summer) (Hietala *et al*. 2013).

**Fig. 3.**
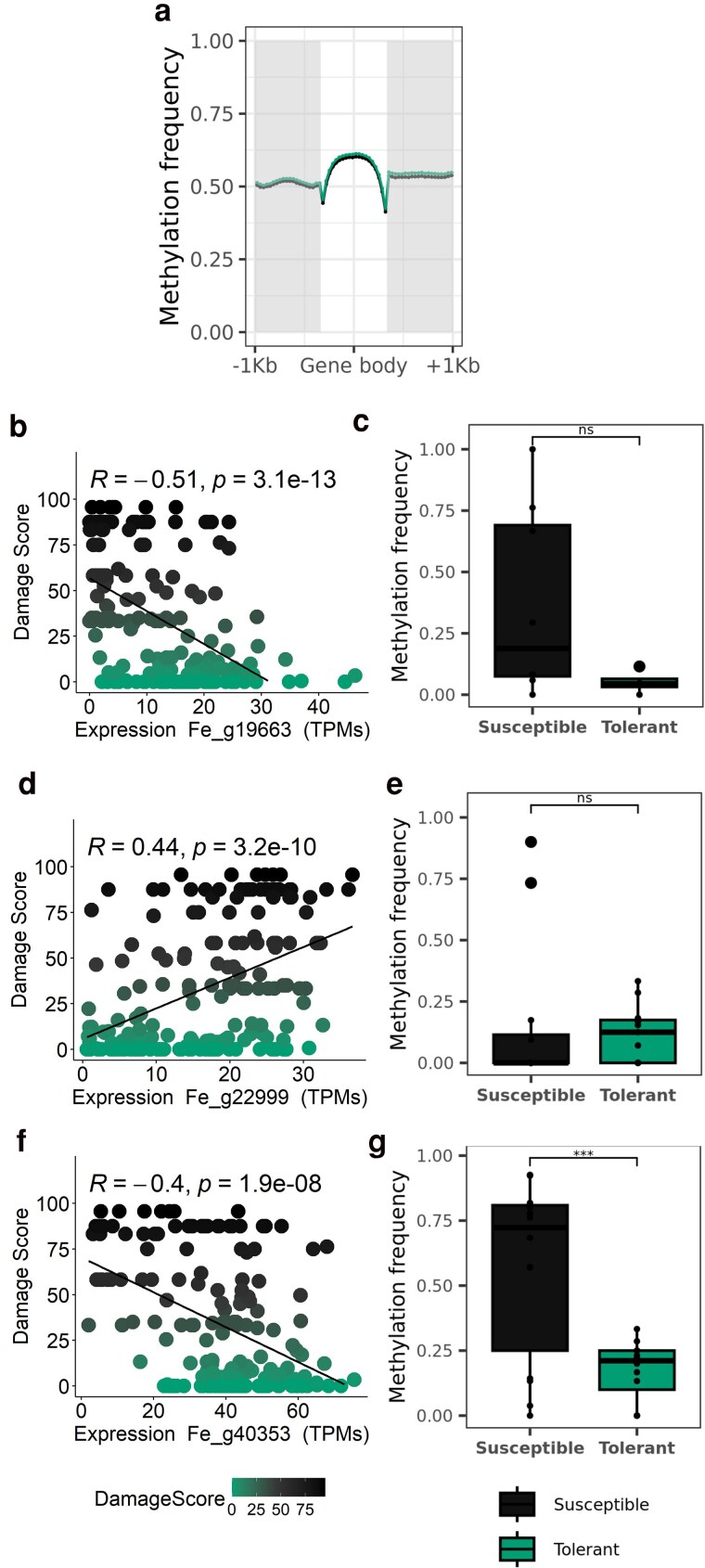
Expression and DNA methylation of genes in the new *F. excelsior* genome. a) Mean methylation frequency across all the genes in the new genome, according to the gene body and upstream and downstream regions (1 kbp each). Mean methylation was calculated in 20 intervals of the length of the gene body or the selected upstream and downstream regions. In green the results of tolerant tree to ash dieback and in black the susceptible tree. b, d, e) Spearman correlation between the expression of the GEMs Fe_g19663, Fe_g19663, and Fe_g40353, identified as SOC1, SVP-like, and SOC1, respectively, and the damage score in each tree (green for the tolerant tree to ash dieback and black for the susceptible tree). c, e, g) Methylation frequency differences between the susceptible and tolerant trees in the upstream region (1 kbp) of the GEMs: Fe_g19663 (*P* = 0.054), Fe_g19663 (*P* = 0.7), and Fe_g40353 (*P* = 0.0008). Stars indicate significant differences. The same colors as in a) were applied.

Correlations between phenology and disease susceptibility have been already observed in other trees such *Quercus agrifolia* against *Phytophthora ramorum* (Dodd *et al*. 2008) or *Ulmus minor* trees against Dutch elm disease (Domínguez *et al*. 2022). Mckinney *et al*. (2014) found a moderate correlation in Danish and Sweden ash trees between earlier flushing and more resistant genotypes, which can explain this relationship with MADS-box genes. Therefore, we assessed if the different gene expression levels of the top 3 MADS-box (Fe_g19663, Fe_g22999, and Fe_g40353) correlated (Spearman correlation) with the damage score. We showed that the expression of SOC1-like Fe_g19663 and Fe_g40353 was negatively correlated with damage, whilst expression of Fe_g22999 was positively correlated (Fig. 3). Considering that the RNA samples were collected during the time of flushing (Harper *et al*. 2016), susceptible trees, with higher SVP-like expression, might be expected to exhibit delayed phenology, whilst tolerant trees might flush and senesce earlier in the year due to reduced expression of SVP-like (Fe_g22999) and increasing expression of SOC1-like (Fe_g19663 and Fe_g40353) genes.

Epigenetic regulation of DORMANCY-ASSOCIATED MADS-BOX (DAMs) has also been reported, with DNA methylation found in the promoter of MADS associated with transcript repression (Rothkegel *et al*. 2017). However, until now, it has not been assessed whether there are differences in DNA methylation between susceptible and tolerant trees to ash dieback. As a preliminary investigation, we assessed DNA methylation in 2 trees with different degrees of susceptibility. More than 8.75 and 9.05 billion bases (350,247,763 and 35,841,634 reads, N50 = 33,797 and 25,093 and Q20 = 14 and 15%) constituted the tolerant and susceptible ash samples, respectively. The coverage was >10 × for each sample (tolerant sample average read length was 248.3 and standard deviation = 71,831.2; susceptible sample average read length was 252.4 and standard deviation = 73,683.0). There were a total of 187,439,544 and 178,011,401 CpG sites and an average methylation frequency of 67.2 and 66.1% in the susceptible and tolerant trees, respectively. When looking at the methylation frequency across all genes (Fig. 3a), no differences were seen between trees. However, we observed a significantly higher overall DNA methylation profile in the promoter of the gene coding for a SOC1-like protein in the susceptible tree [after removing outliers mean was 36.8% vs 5.2% in Fe_g19663 (*P* = 0.0541) and 56.9% vs 17.3% in Fe_g40353 (*P* = 0.00081) for susceptible and tolerant, respectively; Fig. 3c and g], which might result in lower expression of this gene (Fig. 3b and f). The opposite pattern was seen in SVP-like (Fe_g22999), with slightly but no significantly higher DNA methylation in the tolerant line [after removing outliers 15.8% vs 12.0% for susceptible and tolerant (*P* = 0.7), respectively; Fig. 3e], which again could result in lower expression of this gene (Fig. 3d). Overall, the DNA methylation analyses showed variation in DNA methylation between a tolerant and susceptible tree, which consequently might regulate the expression of markers associated with different ash dieback susceptibility. However, to confirm epigenetic regulation of these genes, larger groups of susceptible and tolerant trees must be assessed, followed by analysis to identify differentially methylated regions.

## Conclusions

In summary, we have obtained a more contiguous ash genome which has allowed us to confirm the importance of phenology timing to combat ash dieback and identified 175 GEMs associated with disease damage scores. The new GEMs included several MADS-box genes, such as SOC-1, SVP-like proteins, and Dormancy MADS-box JOINTLESS, that showed differences in expression between susceptible and tolerant trees potentially regulated by differences in DNA methylation in the promoters. Identifying the loci responsible for tolerance to ash dieback could help in breeding programs of ash trees and combat this devastating pathogen.

## Supplementary Material

jkaf053_Supplementary_Data

## Data Availability

The whole data of this project can be found under the BioProject PRJNA865134 being the *F. excelsior* genome BioSample SAMN30100368, genome JANJPF000000000. Methylation of this genome can be found in the GEO under the number GSE214553. Nanopore raw sequencing reads, and methylation profiles of the tolerant and susceptible samples are available in GSE214552, methylation profile of the new genome and salmon counts of the Danish population against the new genome (mRNA-Seq sequenced by Harper *et al*. 2016) are available at GEO under the number GSE214551. Chloroplast and mitochondrial genomes are available at GenBank OP360910 and OP360911 to OP360929, respectively, whilst the sequence of the 3 MADS-box is available at GenBank with accessions OP133138, OP133139, and OP133140. The new genome and annotations, the methylation profiles and the variant calling file can also be retrieved from https://webfiles.york.ac.uk/Harper/Fraxinus_excelsior/ or the GSA figshare at https://doi.org/10.25387/g3.28541393, and the scripts can be found at https://github.com/sfortega/Ash_genome. Supplemental material available at G3 online.
