# Supplementary material for: Fraxinus excelsior updated long-read genome reveals the importance of MADS-box genes in tolerance mechanisms against ash dieback
Source: G3 (Bethesda). 2025 Mar 20;15(5):jkaf053. doi: 10.1093/g3journal/jkaf053 (PMC12060229; doi:10.1093/g3journal/jkaf053)
Supplement: jkaf053_Supplementary_Data [file jkaf053_supplementary_data.zip › Supplemental_Material_Legends_G3-2024-405603.docx]

**Supplemental Material Legends**

**Table S1.** Gene ontology enrichment of 4804 gene models in the new *F. excelsior* assembly that did not have high similarity with any of the cds models in the *F. excelsior* genome obtained by Sollars et al. (2016).

**Table S2.** Synima results using Orthofinder and the coding sequence of *F. excelsior* and *F. pennsylvanica* genes.

**Table S3**. Uniquely mapping rates using STAR of the reads of the Danish population.

**Table S4**. PSIKO population structure.

**Table S5**. Gene expression markers result with FDR adjusted.

**Figure S1. Plots of the new *F. excelsior* genome. A**. Chord diagram showing similarity between *F. excelsior* contigs and *F. pennsylvanica* chromosomes. Each line represents regions with high similarity (>90%) between the sequence of *F. excelsior* contigs and *F. pennsylvanica* chromosomes. Each chromosome is represented in a different color*.* **B**. Plot of the F. excelsior genome. The circles represent (external to internal): contigs location (in the + strand in light blue and – strand in dark blue), genes (in the + strand in light red and – strand in dark red), GC content, methylation frequency, LTRs (in the + strand in orange and – strand in black), TIRs (in the + strand in yellow and – strand in black) and the MADS-box location.
