# Supplementary material for: Fraxinus excelsior updated long-read genome reveals the importance of MADS-box genes in tolerance mechanisms against ash dieback
Source: G3 (Bethesda). 2025 Mar 20;15(5):jkaf053. doi: 10.1093/g3journal/jkaf053 (PMC12060229; doi:10.1093/g3journal/jkaf053)
Supplement: jkaf053_Supplementary_Data [file jkaf053_supplementary_data.zip › Supplementary_text1.docx]

**Supplementary text:**

**Material and methods**

**Chloroplast and mitochondria assembly**

Organelle reads were extracted in a similar way as reported by Syme et al., (2021). Chloroplast reads were initially extracted from the raw reads by mapping with MINIMAP2 (Li 2018) with the full chloroplast genome of *Fraxinus* *angustifolia* (GenBank accession number MH817870.1) a circular plastidome of 155574 bp. SAMTOOLS (Li et al. 2009) were used to extract the mapped reads and used as input with CANU assembler (Koren et al. 2017). Finally, the assembled reads were polished with RACON version 1.4.20 and MEDAKA version 1.0.3. (Vaser et al. 2017)and the polished reads were used again as the query of the raw reads to obtain the full dataset of chloroplast reads.

Unmapped chloroplast reads of the second round were used to map 36 *Fraxinus ornus* mitochondria genes (MG457288.1, MG431798.1, MG431788.1, MG431778.1, MG431768.1, MG431758.1, MG431748.1, MG431738.1, MG431728.1, MG431718.1, MG431708.1, MG431698.1, MG431688.1, MG431678.1, MG431668.1, MG431658.1, MG431648.1, MG431638.1, MG431628.1, MG431618.1, MG431608.1, MG431598.1, MG431588.1, MG431578.1, MG431568.1, MG431558.1, MG431548.1, MG431538.1, MG431528.1, MG431518.1, MG431508.1, MG431498.1, MG431488.1, MG431478.1, MG431468.1, MG431458.1). The mitochondria was assembly following the same approach than with the chloroplast. QUAST and SEQKIT stats were used after each round to check the completeness of both organelle genomes.

Both chloroplast and mitochondria genomes were annotated using MPI-MP Chlorobox GeSeq tool (Tillich et al. 2017). The Chlorobox set up for the chloroplast included annotations of plastid inverted repeat (IT), the trans-spliced rps12, support annotation by Chloë, tRNA-scan-SE v2.0.7 (Organellar tRNA, universal genetic code and score cut-off of 15) and used as Reference the chloroplast land plants+rRNA

Mitochondrion genome was annotated using 233 mitochondria genomes as 3^rd^ Party references (accessed 26.03.2022) as a linear genome, keeping all annotation, BLAT searches with 25% protein identity and 70% rRNA, tRNA, DNA identity, and the tRNAscan-SE v2.0.7 tool for annotating tRNAs (Universal genetic code and score cut-off of 15). Only one round of mitochondria was used to obtain the assembly, but a second round using the medaka polished as input for MINIMAP2, was used to remove more reads mapping to this organelle before assembling the genome.

Methylation was called with NANOPOLISH for both organelles and the frequency of methylated reads was calculated using the PERL script calculate_methylation_frequency.py

**Results**

**Chloroplast and mitochondria genome assemblies**

After the first round, a total of 8,330 plastome reads were extracted with a coverage of 200.04x (3200816 bases). MEDAKA polishing produced an assembly with a total length of 180,159. After the second round, a total of 9,387 reads were mapped (36,000,193 bases, 200 times coverage) and one single contig was assembled by CANU, RACON and MEDAKA (total length was 192837, 37.54% G+C content).

Instead, the mitochondria assembly reached a total size of 592,457 bp divided into 19 contigs, with a GC content of 44.65% (N50 59,582 divided into 19 contigs (minimum length was 3,680 bp and maximum 116,956 with average 31,209.7). However, the second round of CANU, RACON and MEDAKA produced an assembly of more than 900 kbp, and we decided to keep the assembly of the first round as the final mitochondria genome.

A total of 218 gene models (244 exons and 37 introns) and 11 rRNA models were annotated in the chloroplast, whilst the mitochondrion genome was annotated with 1824 gene models (169 exons, 1454 CDS, 97 introns), 168 rRNAs and 298 tRNAs.

**References**

Koren S, Walenz BP, Berlin K, Miller JR, Bergman NH, Phillippy AM. 2017. Canu: scalable and accurate long-read assembly via adaptive k-mer weighting and repeat separation. Genome Res. 27:gr.215087.116. doi:10.1101/GR.215087.116.

Li H. 2018. Minimap2: pairwise alignment for nucleotide sequences. Bioinformatics. 34:3094–3100. doi:10.1093/BIOINFORMATICS/BTY191.

Li H, Handsaker B, Wysoker A, Fennell T, Ruan J, Homer N, Marth G, Abecasis G, Durbin R. 2009. The Sequence Alignment/Map format and SAMtools. Bioinformatics. 25:2078–2079. doi:10.1093/BIOINFORMATICS/BTP352.

Syme AE, McLay TGB, Udovicic F, Cantrill DJ, Murphy DJ, McLay TGB, Udovicic F, Cantrill DJ, Murphy DJ. 2021. Long-read assemblies reveal structural diversity in genomes of organelles – an example with *Acacia pycnantha*. GigaByte. 2021:1–23. doi:10.46471/GIGABYTE.36.

Tillich M, Lehwark P, Pellizzer T, Ulbricht-Jones ES, Fischer A, Bock R, Greiner S. 2017. GeSeq - versatile and accurate annotation of organelle genomes. Nucleic Acids Res. 45(W1):W6–W11. doi:10.1093/NAR/GKX391.

Vaser R, Sović I, Nagarajan N, Šikić M. 2017. Fast and accurate de novo genome assembly from long uncorrected reads. Genome Res. 27:737–746. doi:10.1101/GR.214270.116/-/DC1.
