# Supplementary figures and images for: Fraxinus excelsior updated long-read genome reveals the importance of MADS-box genes in tolerance mechanisms against ash dieback
Source: G3 (Bethesda). 2025 Mar 20;15(5):jkaf053. doi: 10.1093/g3journal/jkaf053 (PMC12060229; doi:10.1093/g3journal/jkaf053)

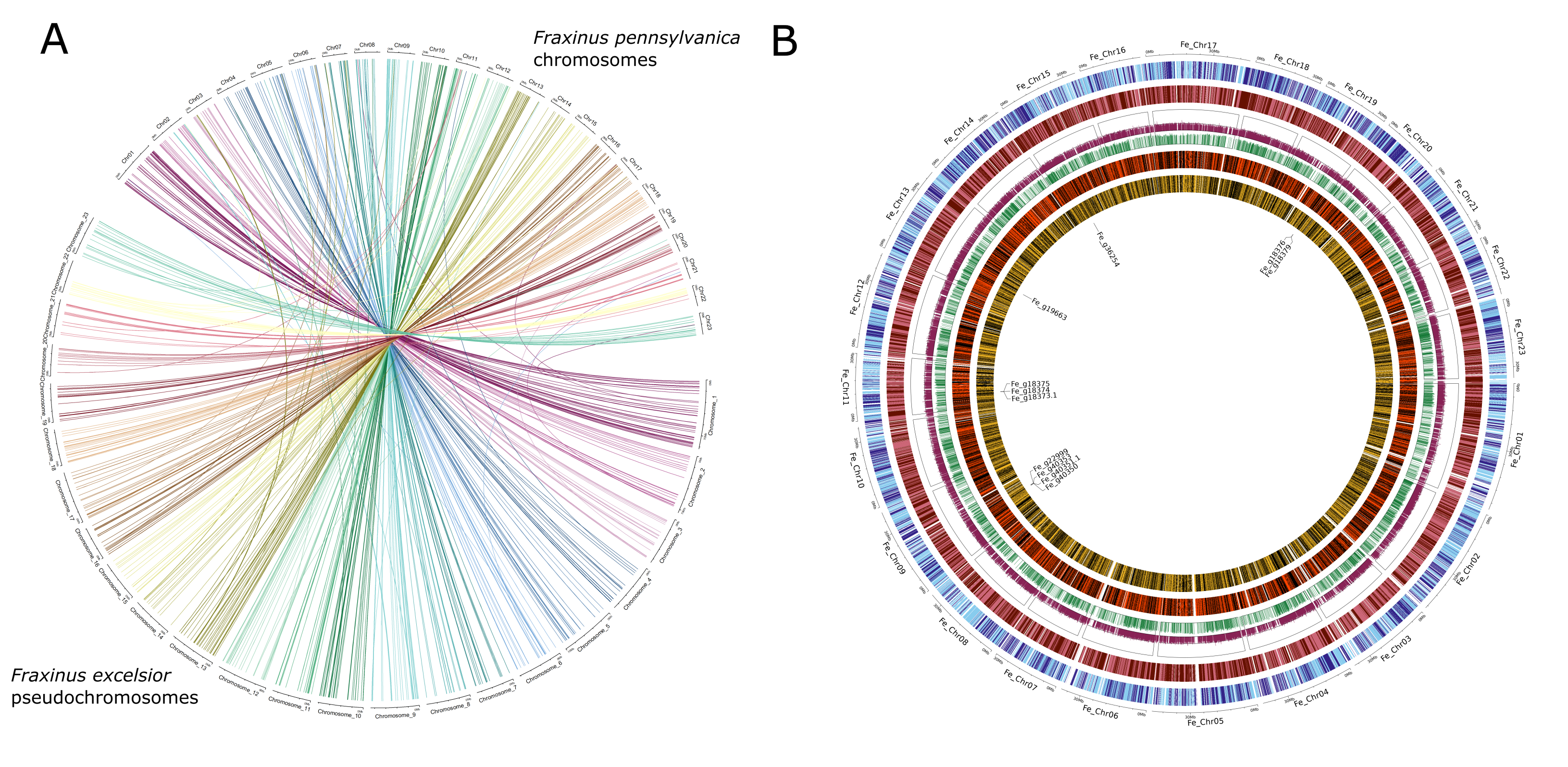

Supplement: jkaf053_Supplementary_Data [file jkaf053_supplementary_data.zip › Figure_S1_G3-2024-405603.png]
